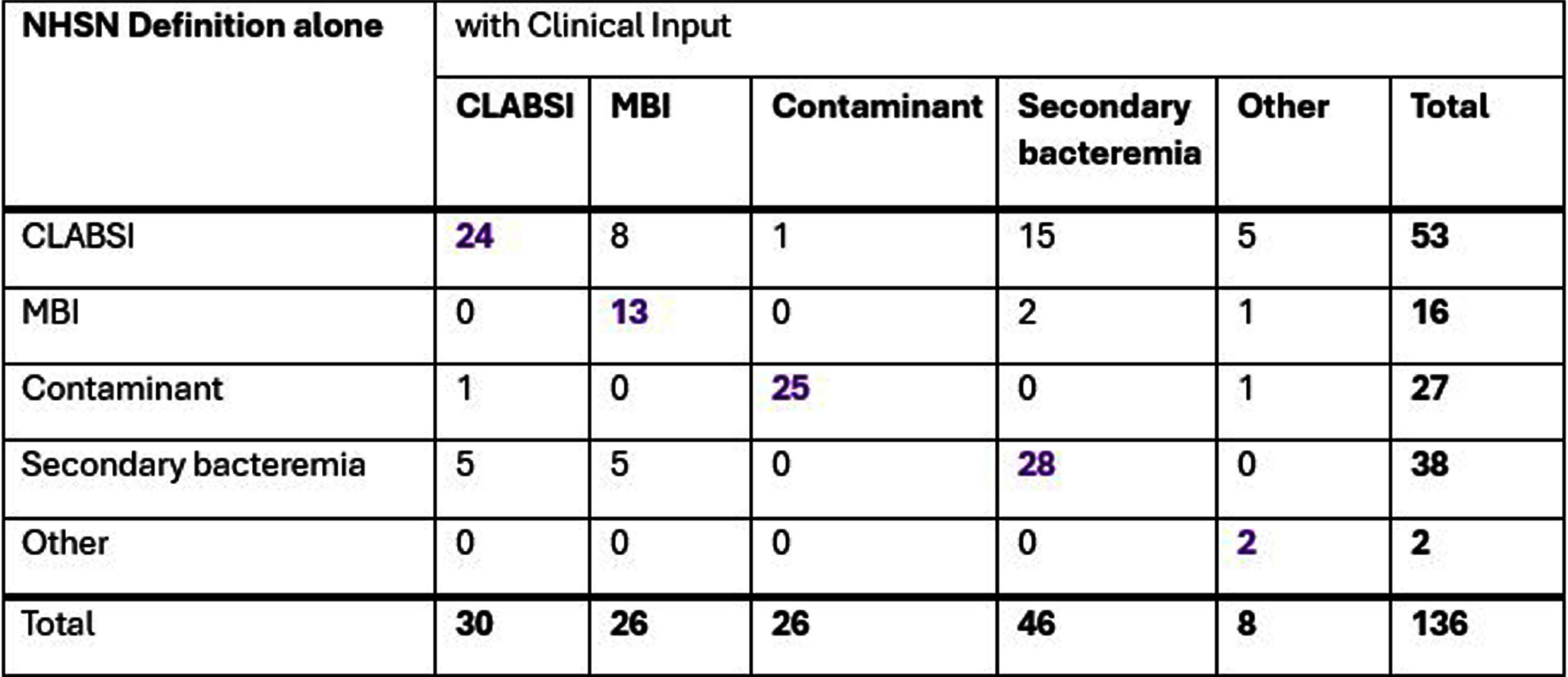# Classifying pediatric central line-associated bloodstream infections: finding meaning by comparing surveillance to clinical definitions

**DOI:** 10.1017/ash.2025.290

**Published:** 2025-09-24

**Authors:** Monica Monteon, Nada Harik, Annette Lee, Michelle Liberty, Brigid Blouin, Xiaoyan Song

**Affiliations:** 1 Children’s National Hospital

## Abstract

**Background:** Central line-associated bloodstream infections (CLABSIs) are monitored in U.S. hospitals using the National Healthcare Safety Network (NHSN) surveillance definitions. This standardization has enabled interfacility comparisons of CLABSI rates and established CLABSIs as a nationally recognized healthcare quality and patient safety indicator. Since CLABSI prevention efforts focus on infections meeting the NHSN definition, fewer resources are allocated to address other bacteremia sources, potentially missing opportunities for improvement. **Methods:** The review included hospitalized patients with an eligible central line and ≥1 positive blood culture on hospital day ≥ 3 in 2024. Trained infection preventionists (IPs) applied the NHSN surveillance definitions to classify positive blood cultures. IPs then gathered clinical information by reviewing the patients’ medical history, interventions, imaging tests, antimicrobial treatments, and direct caregiver engagement, used it to determine the likely clinical sources for bacteremia, and classified them according to NHSN categories. The concordance in classifying positive blood cultures using the NHSN surveillance definition alone versus with clinical input were compared. **Results:** Of the 136 eligible cases that IPs reviewed in 2024, 92 (67%) had concordant classifications as CLABSI (24), mucosal barrier injury (MBI) (13), secondary bacteremia (28), contaminant (25), or other (2). Of the 29 CLABSIs that met only the NHSN surveillance definition, 15 were associated with a clinical secondary source, 8 with a clinical MBI episode, 5 as continuation of previous infection or present on admission, and 1 as clinical contaminant. The 83 non-CLABSI bacteremia included 38 infections at other sites and 27 contaminants. **Conclusion:** Our analysis suggests that using NHSN surveillance definitions results in significant overreporting of CLABSIs in pediatric patients. Overreporting may be due to factors unique to the pediatric population, such as the inability to communicate clinical symptoms and the normal physiologic lack of signs needed to meet NHSN definitions. A focus on all BSIs could provide a greater benefit towards hospital harm reduction activities by focusing on the likely true source of bacteremia. Compared to CLABSIs, patient harm from contaminated blood cultures and infections with secondary bacteremia may be more prevalent and require a greater focus on prevention.

**Figure:**
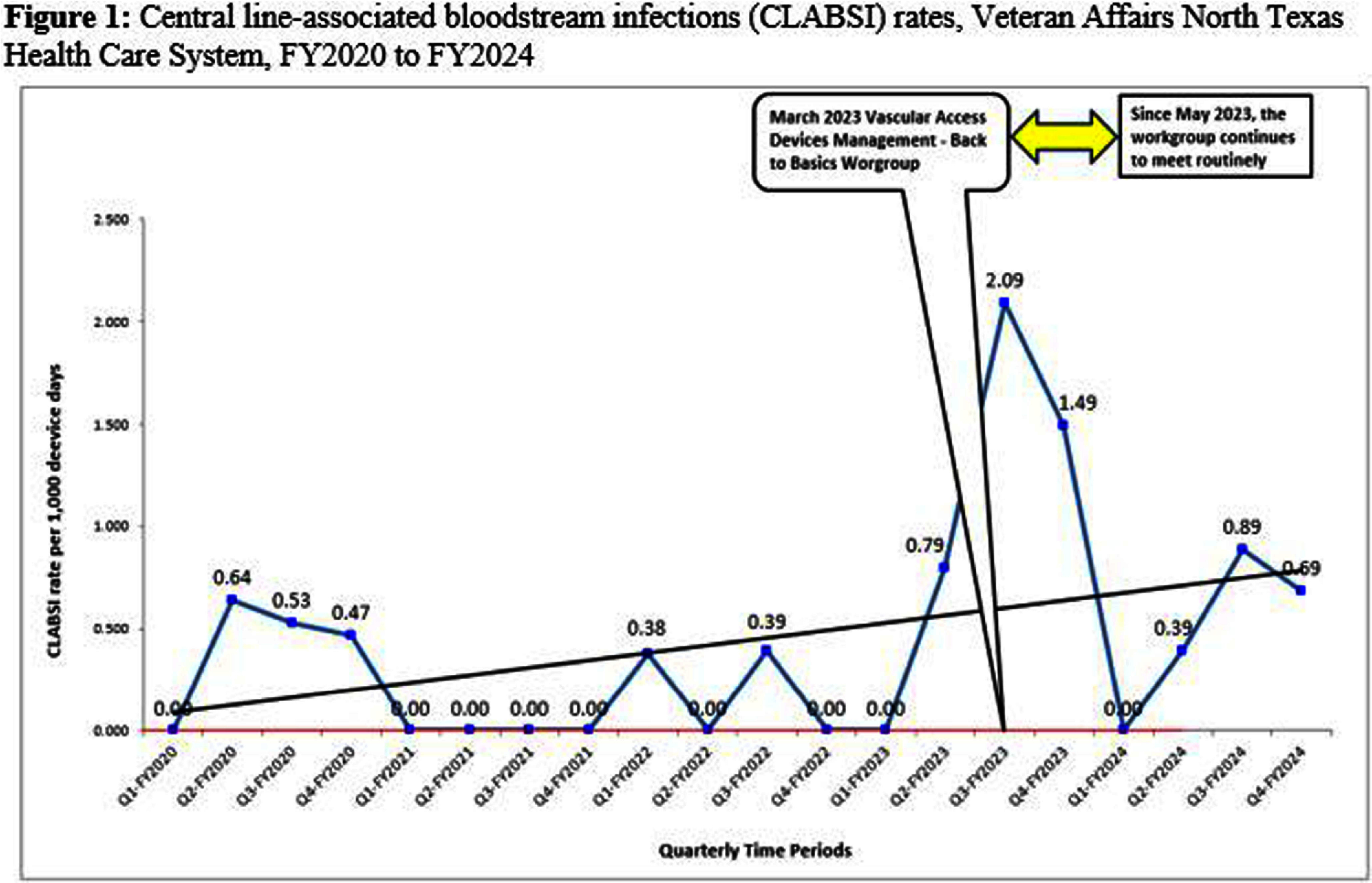

**Figure:**